# Boneless occiput and awake fibreoptic intubation in lateral position

**DOI:** 10.4103/0019-5049.65355

**Published:** 2010

**Authors:** Keshav Goyal, Girija P Rath, Tumul Chowdhury, Charu Mahajan

**Affiliations:** Department of Neuroanaesthesiology, All India Institute of Medical Sciences, New Delhi, India

Sir,

Tracheal intubation with a patient in a lateral position is not a routine procedure. The use of a laryngeal mask airway (LMA) has been suggested for difficult airway situations with a patient in a lateral position.[1] Tracheal intubation using fibreoptic bronchoscope remains an alternative. However, awake fibreoptic intubation with a patient placed lateral has never been described. Nathanson and colleagues suggested such a concept in a manikin.[2] An 18-year-old male with operated ‘suboccipital intra-diploic subarachnoid cyst’ [Figure 1] was scheduled for ventriculo-peritoneal shunt (VP) surgery. He underwent two different surgeries under general anaesthesia (GA), 10 days prior. In the first procedure, craniotomy and de-roofing of the suboccipital cyst was carried out. Four days later, the patient developed intra-diploic pseudomeningocoele with the sub-galeal collection of CSF for which a sub-galeo-peritoneal shunt was inserted. In both the occasions, the airway was secured by fibreoptic orotracheal intubation under GA. There was difficulty in mask ventilation during the second surgery, as the patient was placed lateral. Six days later, the sub-galeo-peritoneal shunt was blocked; hence, VP shunt insertion was planned. The patient had restricted extension of neck after the first surgery which comprised cranioplasty, and fixation of the suboccipital bone and cervical spine with a mini-plate and screw. The mouth opening was less than two fingers’ breadth with a mallampatti grade IV. The patient was nursed in a lateral position as the occipital part of the skull was boneless, and because of his comfort on the right side. There occurred bradycardia, whenever the patient was placed supine. Possibly, the external pressure was transmitted to the brainstem or intracranial pressure increased, resulting in haemodynamic disturbances. Hence, it was planned to intubate the trachea with the patient awake, using a fibreoptic bronchoscope in the right lateral position. The patient was very cooperative. The oropharyngeal airway was anaesthetized using 10% lignocaine spray. Fibreoptic intubation was attempted by a spray-as-you-go (SAYGO) technique, using a 2% lignocaine solution. Inspite of a difficult spatial orientation, the fiberscope was negotiated into the trachea. Intubation was successful, albeit, with difficulty in railroading the tracheal tube (Portex, Smiths Medical International Ltd., Kent, UK; size 8.0 I.D.). The total intubation time was 110 s. This was followed by the induction of anaesthesia with a standard technique. Rest of the surgical course was uneventful. At the end of the surgery, the trachea was extubated after reversal of the residual neuromuscular blockade.

**Figure 1:**
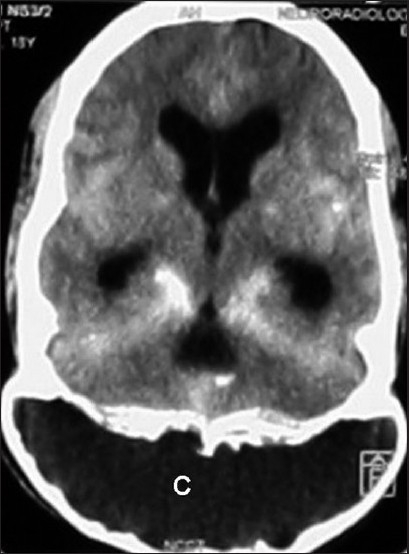
MRI of the skull (T1-weighted image) shows a suboccipital intradiploic subarchnoid cyst (C)

Securing an airway in patients with anticipated difficult intubation is always a challenge. To carry out this manoeuvre in a lateral position seems to be more complicated, especially when the patient is awake. The left lateral position results in the deterioration of the laryngoscopic view in 35% of patients, without any improvement.[2,3] Anaesthetic trainees found tracheal intubation in the left lateral position more difficult than in supine.[2] However, the left lateral position is preferred to the right for intubation as it prevents laryngeal structures from collapsing.[3] Our patient was comfortable in the right lateral position; hence, intubation was performed in that position. The use of a large donut-shaped pillow could have kept the patient supine, but the problems of boneless occiput might not have been ruled out. Anaesthetizing the airway with a bilateral superior laryngeal nerve block, trans-tracheal injection of local anaesthetic is feasible, but, difficult to be carried out owing to improper position. Hence, the SAYGO technique was preferred. We feel that it is necessary to learn the art of awake fiberoptic intubation in patients placed lateral. This manoeuvre may be particularly useful while securing airways in neurosurgical patients with a large encephalocele and boneless occiput, as in this case.

## References

[1] Chen CH, Lin CC, Tan PP (1995). Clinical experience of laryngeal mask airway in lateral position during anesthesia. Acta Anaesthesiol Sin.

[2] Nathanson MH, Gajraj NM, Newson CD (1994). Tracheal intubation in a manikin: Comparison of supine and left lateral positions. Br J Anaesth.

[3] Adachi YU, Satomoto M, Higuchi H (2002). Fiberoptic orotracheal intubation in the left semi lateral position. Anesth Analg.

